# Editorial: Global Population Aging - Health Care, Social and Economic Consequences

**DOI:** 10.3389/fpubh.2018.00335

**Published:** 2018-11-20

**Authors:** Seiritsu Ogura, Mihajlo Michael Jakovljevic

**Affiliations:** ^1^Faculty of Economics, Hosei University, Tokyo, Japan; ^2^Global Health Economics and Policy, Faculty of Medical Sciences, University of Kragujevac, Kragujevac, Serbia; ^3^Division of Health Economics, Lund University, Lund, Sweden

**Keywords:** population aging, demographics, gLobal, health economics, policy

Population Aging or Third Demographic Transition nicknamed as well “The Silver Cunami” became a virtually global phenomenon in the second half of XX century (1). Purpose of this Research Topic was to gather an array of submissions with the plausible goal of depicting its health care related and socioeconomic consequences. A total of four articles excluding this Editorial have been published inclusive three full length original research papers and one book review. Base affiliations of the authors spread across academia and governmental authorities while these universities and institutes were situated in the US, China, Australia, Germany and Serbia.

While there had been some isolated periods of fertility declines in Europe in the last two centuries, the phenomenon of the so called Third Demographic Transition became broadly recognized in academic literature and UN reports mostly over the past three decades (2). It consists of falling female fertility, improved early childhood survival, extended longevity, and ultimately the rapid growth in the proportion of the elderly population in a society. Among the major underlying causes the following are listed: growing living standards, improvements in medical services, public health and public education, and sexual revolution and consequent absorption of unpaid female labor into the labor markets (3). Interdependency of personal savings and labor force participation of the elderly, and social security wealth: A time series analysis. Appl. Econ. 24, 379–388). In the process, the families undergo significant changes in their social role; large or multi-generation families dissolve into nuclear families, keeping the reproduction, consumption, education, and health production functions, but losing most of the functions for production and intergenerational resource transmission (4).

The transition to an aged society brings serious financial challenges to all institutions in any economy. Even most developed countries that had foreseen these problems coming for two decades are still struggling to find money to pay for the bulging retirement income and health care costs for the growing elderly population (5). Their transition has been made more difficult by the new revolutionary medical technologies, extremely costly, that are targeted to save the life of a relatively small number of patients. To what extent, should public money be spent to save the life of the few, mostly elderly?

Another unsolved health policy problem for the high-income countries is the long-term care for the elderly. With the longevity and differential gender mortalities, we observe not only a swelling population of the very old, overwhelmingly female, and poor, living alone. Only two or three decades ago, they who would have been taken care of by their children or their families, but with family ties weakening, the government is now asked to step in to provide necessary social services for them. How should we finance these costs? How can we preserve incentives for family caregiving? (6).

These problems are no less serious, if not more, for most of the middle-income countries. Compared with the historical experiences of developed countries, their demographic transition is taking place at a much faster pace. It is well known that time to double the proportion of elderly aged above 65 from 7 to 14% of general population share, took a total of 115 years in France while only 21 years in Brazil. While, temporarily these emerging nations may be enjoying huge “demographic dividends,” they are bound to face much larger bills for the retirement income, health care costs and long-term care costs for their disproportionately large elderly populations (7).

With these facts in mind, we believe there are significant gaps in literature, particularly for less recognized middle-income regions; magnitudes of the demographic shocks involved, size of the expected costs of aging, distribution of these costs over generations. Although few developed countries have succeeded in preparing for their demographic shocks, how are these countries preparing for the upcoming aging shocks? Some of the recently published future forecasts on long term health expenditure trends among the leading emerging BRICs economies give a hint of plausible adaptive strategies up to 2025 (7).

Today we have evidence that aging is taking place even among some of the poorest countries, bringing double burdens to their national health system. Still coping with the burden of infectious diseases, injuries, and high neonatal mortality, these communities are facing high toll of chronic non-communicable diseases usually associated with old age in more developed countries (8).

The first piece published in this Topic came from Chinese-Australian collaborative effort was entitled: Dementia Literacy among Community-Dwelling Older Adults in Urban China: A Cross-sectional Study. It has focused on the problem of insufficient knowledge on dementia among the Chinese elderly citizens in the cities and challenge to create educational programs capable of covering this unmet need (Zhang et al.)

Another contribution came from the US and was dealing with the formal and informal home-based medical care service utilization and nursing home service use. It has emphasize to the national authorities “*need to address a diversity of health outcomes and efficiency of services based on providing services to elderly through resource allocation to the different types of long-term care*.” Among the suggested solutions compensation for elderly citizens home-based care was regarded a feasible approach to tackle their serious quality of life issues (Chia-Ching et al.).

German Demographic Institutes jointly with Amsterdam University based group, have conducted a research on suspected renal replacement therapy as a plausible trade-off phenomenon for living longer lives in Western Europe. After a thorough exploration they came to the conclusive remark that extended life expectancy in this region did not statistically correlate with the burden of renal replacement therapy in these same nations. Such claim was grounded in available evidence for 11 Western European countries on 2005–2014, time horizon exploring *post-hoc* analysis of the past ERA-EDTA studies (Peters et al.).

Last of the four pieces was actually a book report prepared on a distinguished public health university text book entitled: The New Public Health 3rd Edition (9). This extensive and encyclopedic contribution encircled almost all relevant disciplines of the science of public health acquiring a strong historical perspective. Authors particularly did pat attention to the segments devoted to the health economics of population aging and its related socio-economic consequences (Jakovljevic et al.).

At the conclusion it is important to mention European Commission funded EUROAGISM project whose outcomes closely deal with employment issues in late career and life stages (10, 11). Based on all aforementioned evidence we may assume that there is growing body of academic literature and funded research on consequences of population aging (12). We hope these efforts will bring new ideas how to adapt to this profound demographic change and invent financially sustainable new models of national health care and social support systems.

## Author contributions

MJ and SO have jointly designed the research question, prepared the manuscript, and revised it for important intellectual content.

### Conflict of interest statement

The authors declare that the research was conducted in the absence of any commercial or financial relationships that could be construed as a potential conflict of interest.
